# A Novel Integrated Inertial Navigation System with a Single-Axis Cold Atom Interferometer Gyroscope Based on Numerical Studies

**DOI:** 10.3390/mi16080905

**Published:** 2025-08-02

**Authors:** Zihao Chen, Fangjun Qin, Sibin Lu, Runbing Li, Min Jiang, Yihao Wang, Jiahao Fu, Chuan Sun

**Affiliations:** 1 College of Electrical Engineering, Naval University of Engineering, Wuhan 430033, China; d24181103@nue.edu.cn; 2 State Key Laboratory of Magnetic Resonance and Atomic and Molecular Physics, Innovation Academy for Precision Measurement Science and Technology, Chinese Academy of Sciences, Wuhan 430071, China; 3 Hefei National Laboratory, Hefei 230088, China; 4 Wuhan Institute of Quantum Technology, Wuhan 430206, China; 5 No. 92038 Unit, The PLA, Qingdao 266109, China; 6 School of Physics, University of Chinese Academy of Sciences, Beijing 100049, China

**Keywords:** cold atom interferometer gyroscope, integrated navigation system, adaptive gradient ascent, Kalman filter

## Abstract

Inertial navigation systems (INSs) exhibit distinctive characteristics, such as long-duration operation, full autonomy, and exceptional covertness compared to other navigation systems. However, errors are accumulated over time due to operational principles and the limitations of sensors. To address this problem, this study theoretically explores a numerically simulated integrated inertial navigation system consisting of a single-axis cold atom interferometer gyroscope (CAIG) and a conventional inertial measurement unit (IMU). The system leverages the low bias and drift of the CAIG and the high sampling rate of the conventional IMU to obtain more accurate navigation information. Furthermore, an adaptive gradient ascent (AGA) method is proposed to estimate the variance of the measurement noise online for the Kalman filter. It was found that errors of latitude, longitude, and positioning are reduced by 43.9%, 32.6%, and 32.3% compared with the conventional IMU over 24 h. On this basis, errors from inertial sensor drift could be further reduced by the online Kalman filter.

## 1. Introduction

An inertial navigation system (INS) is a technology that utilizes an IMU to provide precise navigation solutions, namely, attitude, velocity, and position, using acceleration and rotation rate measurements [1]. Owing to its strong autonomy, high passivity, and inherent covertness, it has been widely used in civilian and military fields, including in land [2], marine [3], aviation [4], and aerospace [5] navigation. However, as a dead-reckoning method based on Newton’s second law, INSs inherently accumulate errors over time, leading to divergence in positioning accuracy [6]. To reduce errors, methods such as global navigation satellite systems (GNSS) [7], acoustic systems [8], and infrared systems [9] are typically integrated. While these approaches effectively enhance INS accuracy, underwater platforms need to transmit sound signals or receive satellite signals by surfacing, which may reduce their concealment. Furthermore, during satellite signal outages, INS/GNSS integrated navigation solutions are not able to achieve the required accuracy. INS accuracy has been improved through extensive research, including that focused on initial alignment [10], damping technology [11], and multi-INS collaboration [12]; however, its further advancement is constrained by the performance of the IMU [13].

Since the first cold atom interferometer (CAI) using stimulated Raman transitions was implemented by the Chu group in 1991 [14], cold atom interferometer gyroscopes (CAIGs) have been developed by numerous research teams [15,16,17,18]. The principle of the CAIG is similar to that of the Sagnac effect in optical gyroscopes, but its theoretical sensitivity surpasses optical gyroscopes by approximately ten orders of magnitude [19]. However, this theoretical advantage of the CAIG is limited in practice. With advancements in cold atom technology, the CAIG has progressed rapidly and been applied to fundamental scientific research, such as in geophysics and metrology [20]. Moreover, as gyroscopes are a core component of IMUs, the CAIG has attracted lots of research interest due to its superior stability and potential for ultrahigh precision [21,22]. However, despite efforts to overcome the limitations of the low dynamic range and low data rate [23,24,25], these limitations persist as key problems for CAIGs in inertial navigation. Currently, most researchers are focused on improving CAI performance using conventional inertial sensors [26,27,28].

This article explores a high-accuracy and high-data-rate inertial sensor through integrating the stability and precision of the CAIG with the sampling rate of the conventional IMU. In addition, to tackle issues of the IMU drift during long-term inertial navigation, an improved Kalman filter with AGA is introduced to enhance the stability and accuracy of the integrated navigation system. Section 2 analyzes the operational principles of CAIGs, and Section 3 details the integrated navigation methodology and the filtering framework. The latter section also describes and validates the simulation experiment method. Conclusions are given in the last section.

## 2. Basic Principles of the Three-Pulse CAIG

This section briefly summarizes the most important and fundamental principles of the three-pulse CAIG. For further details and more rigorous description, readers are referred to [29,30,31,32].

**Figure 1:**
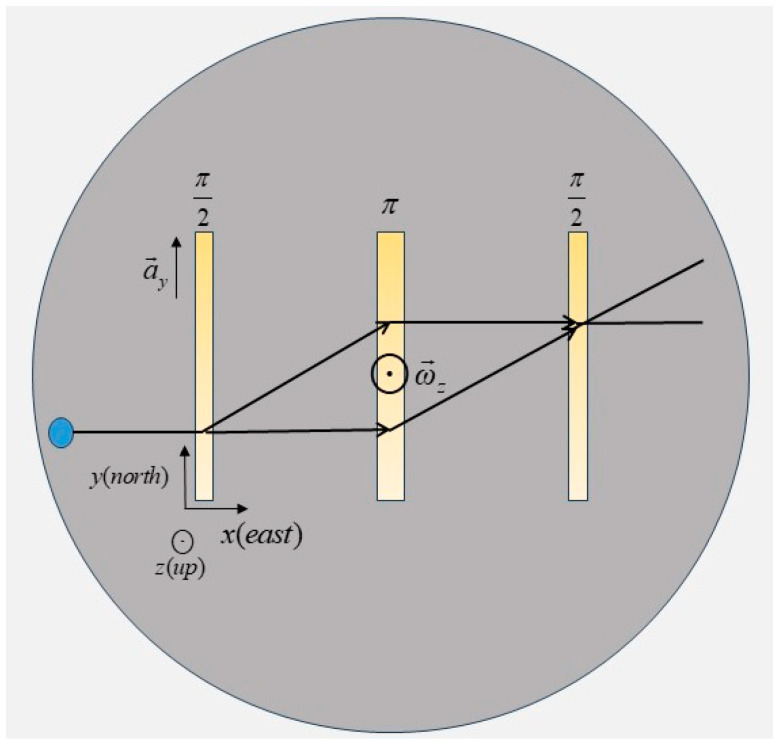
Structure of the CAIG-IMU integrated system. The circular shadow is a stabilized platform, with a CAIG inside. A three-axis gyroscope and accelerometer used by the platform, is regarded as a conventional IMU. The x-axis, y-axis, and z-axis represent the east, north, and up directions, respectively.

The CAIG, by a three-pulse CAI, measures the population of atoms’ states in different energy levels after interference, thereby extracting phase information to calculate inertial quantities (rotation rate and acceleration). Similar to a Mach–Zehnder interferometer, the pulse sequence of the three-pulse atom interferometer is shown above the stable platform in Figure 1. Specifically, ^87^Rb atoms are trapped in a three-dimensional magneto-optical trap (MOT), then they are launched along the positive x-axis at a small polar angle toward the positive z-axis, which ensures that under the influence of gravity, the atoms remain within a common horizontal plane at the time of interaction with the π/2 pulse. After that, atoms are split, reversed, and recombined by three pairs of separated and horizontal Raman pulses (along the north–south direction) separated by time intervals T, which would generate interference. The phase shift in the CAI primarily originates from the phase of the Raman laser acting on the atoms. The phase shift induced by each Raman pulse is expressed as

(1) $$\Delta \phi_{laser}=-k_{eff}a_{y}T^{2}+\frac{2m}{\hbar }\omega_{z}\times A+\Delta \phi^{0}$$

where $k_{eff}$ is the wave vector of the Raman laser, $A=\hbar \left(k_{eff}\times v_{0}\right)T^{2}/m$ is the equivalent interference area, ℏ is the reduced Planck constant, $\Delta \phi^{0}$ is the initial phase difference of the three Raman pulses, T is the interference time between two adjacent pulses, and $v_{0}$ is the velocity of the cold atom [29,30,31,32].

As shown in Equation (1), the phase shift in a three-pulse CAI depends on both acceleration $a_{y}$ and rotation rate $\omega_{z}$. However, the contributions of these two inertial quantities to the phase shift could not be directly distinguished. To address this problem, the CAIG’s design adopts a dual interference loop with opposite-projectile atoms in this article. In the two opposite loops, the acceleration-induced phase shift, $k_{\mathrm{eff}}a_{y}T^{2}$, is equal in magnitude and sign, while the rotation-induced one, $\left(2m/\hbar \right)\cdot \omega_{z}\cdot A$, is equal in magnitude but opposite in sign. By subtracting phase shifts of the two loops, the phase shift related to rotation rate can be successfully extracted; conversely, the acceleration-induced phase shift can be calculated by summing phase shifts of the two loops.

Based on this, we developed a miniaturized CAIG with a size of 787 × 373 × 341 mm^3^, which could measure acceleration and rotation simultaneously using high-resolution dual atom interferometers. Measurements of 40 ng at 518 s and 6.1 nrad/s at 10,880 s were, respectively, achieved for acceleration and rotation [33].

## 3. Single-Axis Integrated Navigation System

An integrated navigation system is explored in this chapter, which ensures the stability and precision of the CAIG with the sampling rate of the conventional IMU. The direction of the navigation frame is defined as east–north–up.

### 3.1. CAIG-IMU Integrated Navigation System

**Figure 2:**
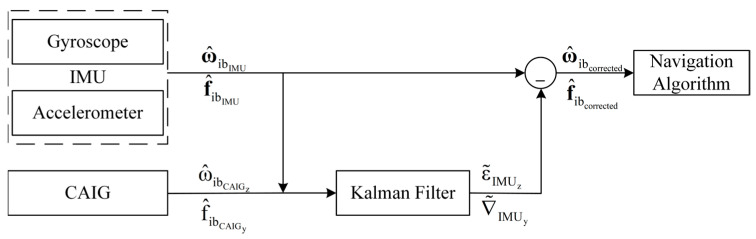
Principle of the integrated CAIG-IMU system.

The integrated navigation system consists of a single-axis CAIG and a stabilized platform, as shown in Figure 1. A three-axis gyroscope and accelerometer used by the stabilized platform, is regarded as a conventional IMU to serve as the primary sensor for inertial navigation algorithms. The stabilized platform provides a stable working environment for the CAIG and ensures that there is no misalignment angle between the conventional IMU frame and the CAIG frame. The CAIG is regarded as an auxiliary sensor to calibrate biases of the conventional IMU. The system workflow is depicted in Figure 2.

As shown in Figure 2, inertial quantities (rotation rate and acceleration) are continuously collected by the conventional IMU for navigation algorithms. When the rotation rate and acceleration could be collected by the CAIG, measurements of the CAIG and the conventional IMU were fed into the Kalman filter to estimate the IMU biases. Therefore, the estimated biases were subtracted from measurements of the conventional IMU to obtain more accurate rotation rate and acceleration, thereby enhancing navigation accuracy.

### 3.2. Bias Estimation of Sensors

For the single-axis integrated navigation system, as described in Section 3.1, because the bias of the conventional IMU is constant in a period of time, the dynamic models of its z-axis gyroscope bias $\varepsilon_{{\mathrm{IMU}}_{z}}$ and y-axis accelerometer bias $\nabla_{{\mathrm{IMU}}_{y}}$ are shown in Equation (2).

(2) $${\dot{\varepsilon }}_{{\mathrm{IMU}}_{z}}=0,{\dot{\nabla }}_{{\mathrm{IMU}}_{y}}=0$$

Then, the state equation of the Kalman filter is established, as shown in Equations (3) and (4),

(3) $$\dot{x}\left(t\right)=F\left(t\right)x\left(t\right)+g\left(t\right)w\left(t\right)$$

(4) $$x(t)={\left[\begin{array}{cc} \varepsilon_{{\mathrm{IMU}}_{z}} & \nabla_{{\mathrm{IMU}}_{y}} \end{array}\right]}^{T},w(t)={\left[\begin{array}{cc} w_{\varepsilon_{{\mathrm{IMU}}_{z}}} & w_{\nabla_{{\mathrm{IMU}}_{y}}} \end{array}\right]}^{T}$$

where $F\left(t\right)$ is the system state matrix, $G\left(t\right)$ is the system noise matrix, and $w\left(t\right)$ represents the process noise vector.

It is well known that the accuracy of the INS is limited by the bias of the IMU. Thus, the true rotation rate and acceleration can be expressed as the following equation:

(5) $$\left\{\begin{array}{l} \omega_{{\mathrm{true}}_{z}}={\hat{\omega }}_{{\mathrm{ib}}_{\mathrm{CAIG}}}-\varepsilon_{{\mathrm{CAIG}}_{z}}={\hat{\omega }}_{{\mathrm{ib}}_{{\mathrm{IMU}}_{z}}}-\varepsilon_{{\mathrm{IMU}}_{z}}\\ f_{{\mathrm{true}}_{y}}={\hat{f}}_{{\mathrm{ib}}_{\mathrm{CAIG}}}-\nabla_{{\mathrm{CAIG}}_{y}}={\hat{f}}_{{\mathrm{ib}}_{{\mathrm{IMU}}_{y}}}-\nabla_{{\mathrm{IMU}}_{y}} \end{array}\right.$$

Equation (5) represents the relationships between measured and true values of the rotation rate and acceleration for the conventional IMU and CAIG, respectively. ω and f are the true values of the rotation rate and acceleration. $\hat{\omega }$ and $\hat{f}$ correspond to measurements of the rotation rate and acceleration from the conventional IMU and CAIG.

Due to $\varepsilon_{{\mathrm{CAIG}}_{z}}\ll \varepsilon_{{\mathrm{IMU}}_{z}}$ and $\nabla_{{\mathrm{CAIG}}_{y}}\ll \nabla_{{\mathrm{IMU}}_{y}}$, merging Equation (5), the fused relationships are derived as

(6) $$\left\{\begin{array}{l} {\hat{\omega }}_{{\mathrm{ib}}_{{\mathrm{IMU}}_{z}}}-{\hat{\omega }}_{{\mathrm{ib}}_{{\mathrm{CAIG}}_{z}}}=\varepsilon_{{\mathrm{IMU}}_{z}}+\eta_{\varepsilon_{{\mathrm{IMU}}_{z}}}\\ {\hat{f}}_{{\mathrm{ib}}_{{\mathrm{IMU}}_{y}}}-{\hat{f}}_{{\mathrm{ib}}_{{\mathrm{CAIG}}_{y}}}=\nabla_{{\mathrm{IMU}}_{y}}+\eta_{\nabla_{{\mathrm{IMU}}_{y}}} \end{array}\right.$$

Therefore, the observation equation of the Kalman filter for the integrated navigation system is formulated as

(7) $$z\left(t\right)=H\left(t\right)x\left(t\right)+\eta \left(t\right)$$

where $z\left(t\right)={\left[\begin{array}{cc} {\hat{\omega }}_{{\mathrm{ib}}_{{\mathrm{IMU}}_{z}}}-{\hat{\omega }}_{{\mathrm{ib}}_{{\mathrm{CAIG}}_{z}}} & {\hat{f}}_{{\mathrm{ib}}_{{\mathrm{IMU}}_{y}}}-{\hat{f}}_{{\mathrm{ib}}_{{\mathrm{CAIG}}_{y}}} \end{array}\right]}^{T}$ is the observation vector, $H\left(t\right)=I_{2\times 2}$ is the measurement matrix, and $\eta \left(t\right)={\left[\begin{array}{cc} \eta_{\varepsilon_{{\mathrm{IMU}}_{z}}} & \eta_{\nabla_{{\mathrm{IMU}}_{y}}} \end{array}\right]}^{T}$ represents the measurement noise vector.

According to Equations (3) and (7), the Kalman filtering equations can be represented as follows.

(8) $$\left\{\begin{array}{l} \dot{x}\left(t\right)=F\left(t\right)x\left(t\right)+G\left(t\right)w\left(t\right)\\ z\left(t\right)=H\left(t\right)x\left(t\right)+\eta \left(t\right) \end{array}\right.$$

Due to the fact that the measurement matrix $H\left(t\right)$ is a unit matrix, the system state is fully observable. The discrete time form of Equation (8) is given by

(9) $$\left\{\begin{array}{l} X_{k}=\Phi_{k,k-1}X_{k-1}+\Gamma_{k-1}W_{k-1}\\ Z_{k}=H_{k}X_{k}+\eta_{k} \end{array}\right.$$

where $\Phi_{k}$ denotes the state transition matrix, $\Gamma_{k-1}$ is the noise distribution matrix, and $W_{k-1}$ and $\eta_{k}$ represent uncorrelated Gaussian white noise sources and are denoted by $Q_{k}$ and $R_{k}$, respectively.

### 3.3. Simulated Experimental Methods and Performance Analysis

**Figure 3:**
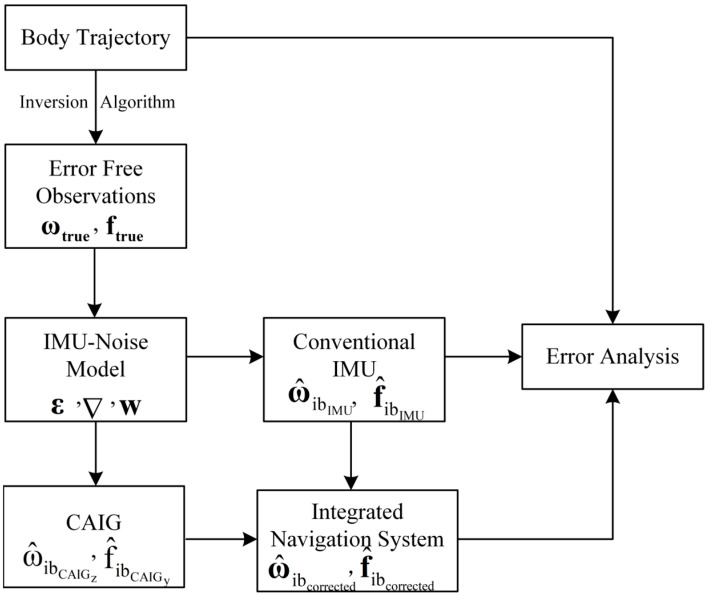
Flowchart of the simulation experiment.

The proposed integrated navigation system is first simulated and verified at the theoretical level in this paper, and dynamic experiments are conducted to further validate the integrated system in future. The experimental workflow is shown in Figure 3.

In the experiment, the long-time motion trajectory of the body can be freely defined; therefore, according to the inverse process of the navigation algorithm [34,35], the error-free rotation rate and acceleration can be derived. Based on the performance of inertial sensors to simulate the integrated navigation system, actual measurements of the rotation rate and acceleration can be obtained by introducing biases and white noises of the conventional IMU and CAIG. As described in Section 3.1 and Section 3.2, the corrected rotation rate and acceleration can be obtained using the Kalman filter. Meanwhile, the measured and corrected rotation rate and acceleration can be input into the navigation algorithm, and then solutions can be separately calculated. Thus, errors can be analyzed by comparing results with the predefined trajectory. The latitude and longitude of Wuhan were set to 30.545° N, 114.355° E, and inertial sensor parameters are listed in Table 1, where parameters of the CAIG are from [33].

**Figure 4:**
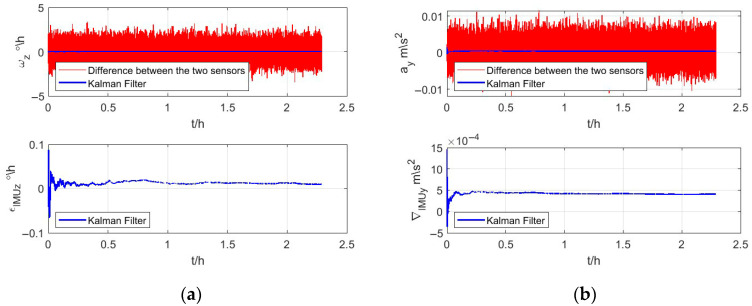
Bias estimations of the conventional IMU. (**a**,**b**) are gyroscope and accelerometer, respectively. In upper subfigures of (**a**,**b**), the red lines are differences between measurements of the conventional IMU and CAIG, and the blue lines are the bias estimations of the gyroscope and accelerometer in a conventional IMU, respectively. The lower subfigures of (**a**,**b**) are amplifications of estimated biases.

According to Table 1 and Section 3.2, the initial value of the Kalman filter can be obtained, and the results of the Kalman filter are shown in Figure 4. The differences between measurements of the conventional IMU and the CAIG are shown in the upper subfigure of Figure 4a,b. The bias estimations of the gyroscope and accelerometer in a conventional IMU are shown in lower subfigures, respectively.

In Table 2, it is shown that biases converge after about one hour, and estimations are highly consistent with parameters of the conventional IMU. The error calculation formula is detailed as Equation (10).

(10) $$\mathrm{Relative}\;\mathrm{Error}=\frac{\left|\mathrm{Estimation}-\mathrm{Actual}\right|}{\mathrm{Actual}}\times 100\%$$

**Figure 5:**
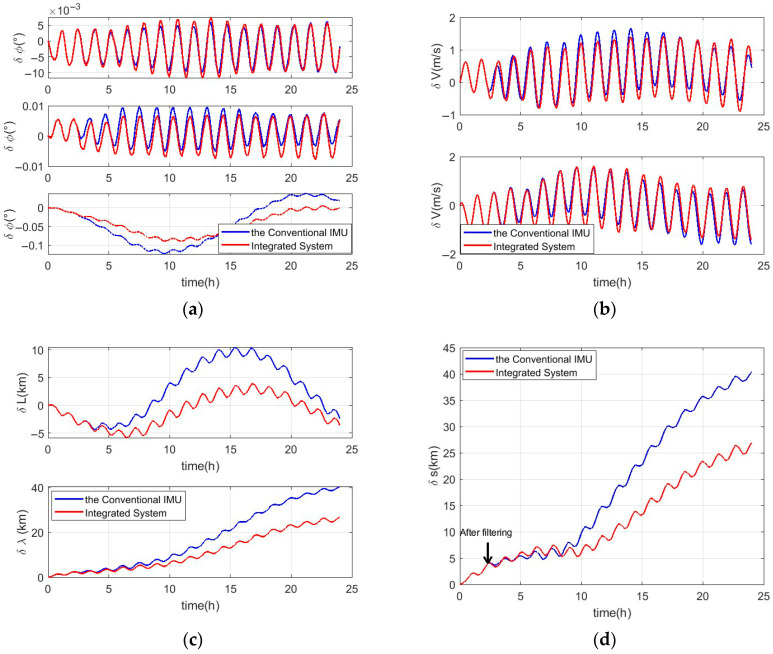
Navigation errors of the conventional IMU and single-axis integrated navigation system. (**a**) The attitude error and images, from top to bottom, are pitch, roll, and yaw, respectively. (**b**) The velocity error. The first image illustrates east velocity, and the other illustrates north velocity. The latitude error is shown in first image (**c**), and the longitude error is shown in second image. (**d**) The positioning error.

After the convergence of the Kalman filter, the estimated bias is subtracted from the conventional IMU’s measurements; then, the more accurate attitude, velocity and position information can be obtained by substituting the corrected measurements into the navigation algorithm. The navigation error analysis is shown in Figure 5 and Table 3, Table 4 and Table 5. In Figure 5, the two results of the conventional IMU and integrated system, respectively, correspond to the blue and red lines.

In Figure 5 and Table 3, Table 4 and Table 5, it can be seen that compared to the conventional IMU, the integrated navigation method effectively improves the INS accuracy, especially in terms of the yaw, latitude, longitude, and positioning, with errors reduced by 25.0%, 43.9%, 32.6%, and 32.3%, respectively. Meanwhile, it can also be found that with the improvement in the accuracy of the z-axis gyroscope and y-axis accelerometer, the precision of the roll and east velocity has improved; however, the north velocity accuracy shows insignificant enhancement, and the pitch error even increases. The main reason for this is that, according to the error differential equation of the INS, the navigation information, including attitude, velocity, and position, is coupled [35]. The yaw error is largely affected by the precision of the z-axis gyroscope. Errors of the roll and east velocity are positively correlated with the yaw error, while the pitch error is negatively correlated with it. Consequently, when the z-axis gyroscope accuracy improves, the roll error and east velocity error decrease, but the pitch error increases slightly. Finally, although the y-axis accelerometer accuracy has improved, the north velocity error is positively correlated with the pitch error, resulting in an insignificant corrective effect on it.

### 3.4. Online Drift Estimation by Adaptive Gradient Ascent

During prolonged operation of inertial navigation systems, despite initial sensor bias compensation through single-run Kalman filtering, inherent limitations in inertial sensor performance inevitably induce progressive measurement bias accumulation due to drift. The navigation accuracy is degraded, necessitating online Kalman filter to mitigate sensor biases. The convergence rate and estimation precision of the filter depend critically on the initial selection of the measurement noise covariance matrix. Conventional approaches utilizing offline variance calculations from full datasets prove infeasible for real-time implementation. To address this problem, an adaptive gradient ascent estimation method is proposed, which could dynamically estimate the initial measurement noise covariance matrix for online Kalman filter.

For gradient ascent estimation, assuming the observed data $z_{i}$ are independent and identically distributed normal random variables following $z_{i}∼Ν\left(0,{\sigma_{i}}^{2}\right)$, the likelihood and log-likelihood functions for a single sample are expressed as follows [36,37]:

(11) $$L\left(\sigma_{i}\left|z_{i}\right.\right)=\frac{1}{\sigma_{i}\sqrt{2\pi }}\exp\left(-\frac{z_{i}^{2}}{2\sigma_{i}^{2}}\right)$$

(12) $$l\left(\sigma_{i}\left|z_{i}\right.\right)=\ln\left(L\left(\sigma_{i}\left|z_{i}\right.\right)\right)=-\frac{1}{2}\ln2\pi -{\ln\sigma }_{i}-\frac{z_{i}^{2}}{2\sigma_{i}^{2}}$$

To ensure $\sigma_{i}>0$, a parameter variable $\theta_{i}=\ln\left(\sigma_{i}\right)$ is introduced, which transforms the estimation of $\sigma ={\left[\begin{array}{cc} \eta_{\varepsilon_{{\mathrm{IMU}}_{z}}} & \eta_{\nabla_{{\mathrm{IMU}}_{y}}} \end{array}\right]}^{T}$ into the estimation of $\theta ={\left[\begin{array}{cc} e^{\eta_{\varepsilon_{{\mathrm{IMU}}_{z}}}} & e^{\eta_{\nabla_{{\mathrm{IMU}}_{y}}}} \end{array}\right]}^{T}$. Equation (12) can be accordingly transformed as follows:

(13) $$l\left(\theta_{i}\left|z_{i}\right.\right)=-\frac{1}{2}\ln2\pi -\theta_{i}-\frac{1}{2}e^{-2\theta_{i}}z_{i}^{2}$$

Taking the derivative with respect to θ yields the gradient

(14) $$g_{i,t}=\frac{\partial l}{\partial \theta_{i}}=-1+e^{-2\theta }z_{i}^{2}=-1+\frac{z_{i}^{2}}{\sigma_{i}^{2}}$$

where $g_{i,t}$ and $\sigma_{i}$ are the gradient and variance of the observation, respectively.

Due to the variance of the rotation rate and acceleration being much closer to zero, direct gradient computation leads to slow convergence or divergence in estimation results. Therefore, the adaptive method is introduced to dynamically adjust the learning rate of the gradient ascent estimation. For further details, readers are referred to [38,39,40]; the most fundamental principles of the algorithm are detailed as follows.

(15) $$m_{t}{=\beta }_{1}m_{t-1}+(1-\beta_{1})g_{t}$$

(16) $$v_{t}=\beta_{2}v_{t-1}+(1-\beta_{2})g_{t}^{2}$$

where $m_{t}$ and $v_{t}$ are the first- and second-moment estimations of the variance; $\beta_{1}$ and $\beta_{2}$ are exponential decay rates for the moment estimates, respectively [38]. Then, corrected moment estimations are computed as follows.

(17) $${\hat{m}}_{t}=\frac{m_{t}}{1-\beta_{1}^{t}},\;{\hat{v}}_{t}=\frac{v_{t}}{1-\beta_{2}^{t}}$$

where ${\hat{m}}_{t}$ and ${\hat{v}}_{t}$ are the corrected first- and second-moment estimations; $\beta_{1}^{t}$ and $\beta_{2}^{t}$ are powers t of $\beta_{1}$ and $\beta_{2}$, respectively. The update principle of the parameter $\hat{\theta }$ is

(18) $${\hat{\theta }}_{i,t+1}={\hat{\theta }}_{i,t}+\alpha \cdot \frac{{\hat{m}}_{t}}{\sqrt{{\hat{v}}_{t}}+\zeta }$$

where α is the learning rate, and ζ is the specified parameter ensuring a non-zero denominator. Meanwhile appropriate parameter values for this study are α=0.1, $\beta_{1}=0.9$, $\beta_{2}=0.999$, and $\zeta =10^{-8}$, respectively.

When the difference of two consecutive results ${\hat{\theta }}_{i,t+1}-{\hat{\theta }}_{i,t}$ is sufficiently low, the recursion algorithm can be stopped. This method enables estimation with minimal samples. The estimated $\hat{\sigma }$ is derived, which initializes the measurement noise covariance matrix for the Kalman filter.

### 3.5. Results and Analyses

**Figure 6:**
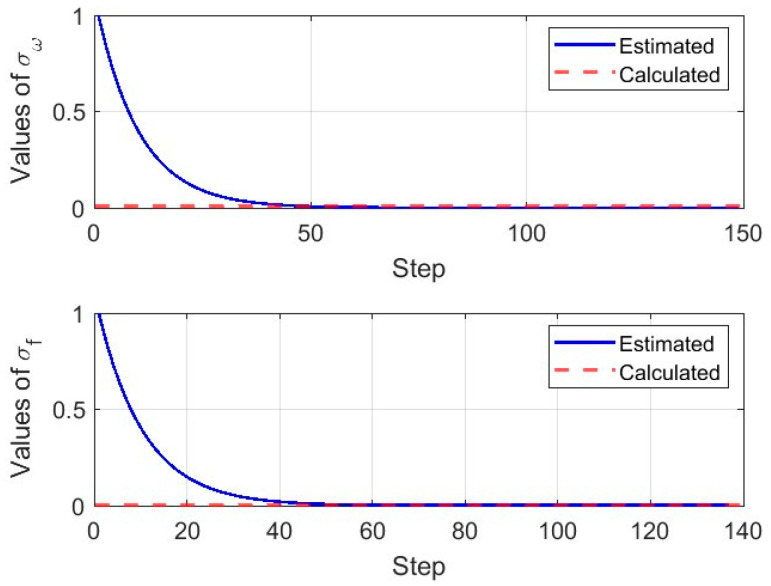
Standard deviation estimations of the adaptive gradient ascent.

**Figure 7:**
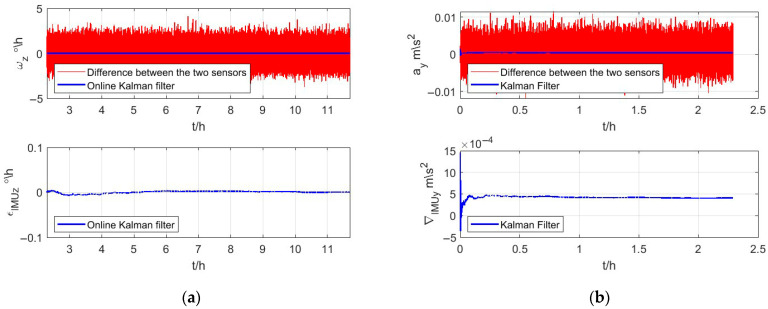
Drift estimations of the conventional IMU. (**a**,**b**) indicate measurements and estimated drift of the gyroscope and accelerometer, respectively.

**Figure 8:**
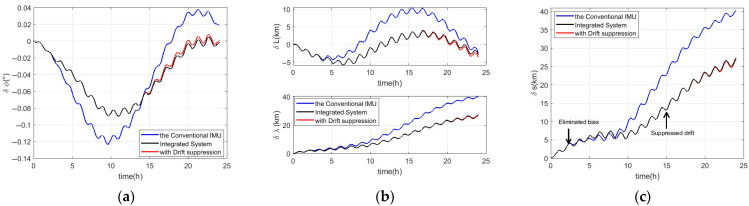
Results of the three kinds of inertial navigation systems. (**a**) The yaw error. In (**b**), the latitude and longitude errors are shown in the upper and bottom images, respectively. (**c**) The positioning error.

On the basis of eliminating sensor bias in Section 3.3, we can further improve navigation accuracy by suppressing drift. Based on the adaptive gradient ascent method, the standard deviation of the corrected measurements after the Kalman filter can be estimated, and the results are shown in Figure 6. The online Kalman filter commences once all standard deviation estimations are acquired, and results of the online Kalman filter are shown in Figure 7. After the convergence of the online Kalman filter, the drift of inertial sensors can be suppressed, and more accurate navigation information can be obtained, which is shown in Figure 8 and Table 6 and Table 7.

The initial standard deviation estimations of the rotation rate and acceleration in observation equation are, respectively, shown in the top and bottom images of Figure 6. The standard deviations of the rotation rate and acceleration converge after 50 iterations, aligning with full-sample calculations. Although there are differences in convergence rates of the standard deviation, which arises from its three-order-of-magnitude discrepancy between rotation rate and acceleration, the time difference to reach the steady-state (≈3 s) is negligible for long-duration inertial navigation systems.

The drift estimations of the gyroscope and accelerometer in the conventional IMU are, respectively, shown in Figure 7a,b. Red lines indicate differences between measurements of the conventional IMU and CAIG, and the estimated drift of the online Kalman filter is shown by a blue line. It could be found that it is feasible to use estimation by AGA as the initial value for the measurement noise covariance matrix, and the online Kalman filter has good stability and fast convergence.

It has been previously discussed that significant improvements in the accuracy of the yaw, latitude, longitude, and positioning occur when the accuracy of the z-axis gyroscope and y-axis accelerometer is enhanced. Therefore, this section only analyzes the four pieces of navigation information. In Figure 8, the blue line is the conventional IMU, the black line represents the integrated navigation system in Section 3.3, and the red line is the integrated navigation system with drift suppression in this section. In Figure 8a and Table 6, it can be seen that although the maximum value of the yaw error has not changed, its root mean square error (RMSE) is smaller. In Figure 8b,c and Table 7, it can be found that on the basis of reducing navigation errors by more than 30% eliminating inertial sensor bias, inertial navigation system errors caused by drift of the IMU can be further reduced by about 1%. The compensation effect for the drift error was not as pronounced, primarily due to the fact that the integrated navigation system in this paper procedure only involved calibrating the bias and drift of one accelerometer and gyroscope using single-axis CAIG, rather than implementing a comprehensive calibration of the full six-axis IMU as a whole.

**Figure 9:**
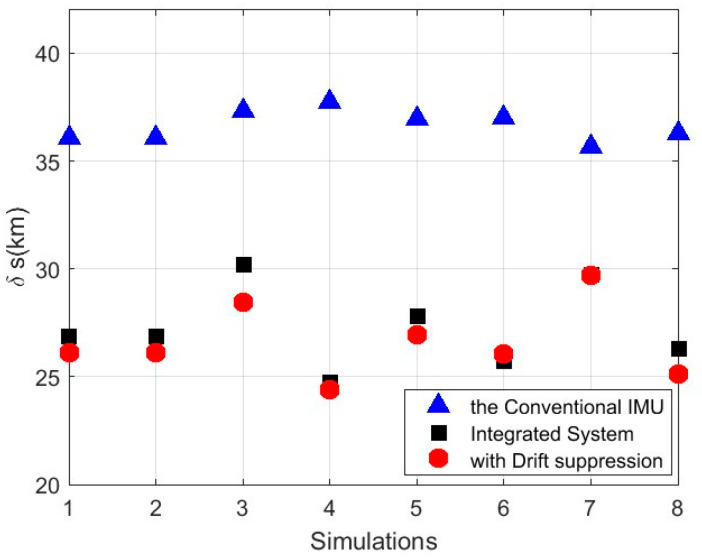
Positioning errors from some additional simulations with different parameters.

To mitigate the randomness of a single simulation run, some additional simulations with different parameters were run. The positioning errors are given in Figure 9. In this figure, it can be seen that the accuracy of the navigation could be significantly improved by the integrated navigation system with the AGA method.

## 4. Discussion and Conclusions

An integrated navigation system combining CAIG with a stabilized platform was designed in this paper. Within this framework, a conventional IMU, comprising gyroscopes and accelerometers in the stabilized platform, serves as the primary sensor for the INS, while the CAIG is a high-precision sensor used to calibrate the bias and drift of the conventional IMU. Furthermore, an adaptive gradient ascent method is proposed, which can utilize minimal data from the conventional IMU and CAIG to obtain the measurement noise covariance matrix for an online Kalman filter to estimate the bias and drift of gyroscopes and accelerometers. Finally, simulation experiments validate the proposed integrated navigation system and hybrid filtering approach.

It was found that the designed single-axis integrated navigation system leverages the low-bias characteristic of the CAIG and the high sampling rate of the conventional IMU. Compared with the conventional IMU, the single-axis integrated navigation system achieves reductions of 43.9%, 32.6%, and 32.3% in latitude, longitude, and positioning errors, respectively. Building upon this enhancement, errors in the inertial navigation system attributable to the conventional IMU drift were further reduced by approximately 1%.

Considering the limited dynamic performance of the CAIG, although the method proposed in this paper has demonstrated promising results in simulation experiments, further analysis and improvement of the integrated navigation system in this paper still require validation through field measurements. Additionally, it was observed that it is effective to use a single-axis CAIG within the inertial navigation system, although substantial optimization remains possible. In response to this, future work will focus on improving the performance of the single-axis CAIG; meanwhile, a dual-axis CAIG is under development, which features bidirectional measurement capabilities for the rotation rate and acceleration across orthogonal axes. We will validate the improvement in the integrated navigation system by adding additional CAIG axes to the measurement and navigation for vehicles, ships, and aircraft.

## Figures and Tables

**Table 1:** Parameters of inertial sensors.

Equipment	Performance	Equipment	Performance
Conventional gyro bias	0.01°/h	Conventional IMU data-rate	200 Hz
Conventional gyro white noise variance	${\left(0.001{}^{\circ}/\sqrt{h}\right)}^{2}$	gyro bias of the CAIG	0.0001°/h
Conventional accelerometer bias	40 μg	Accelerometer bias of the CAIG	40 ng
Conventional accelerometer white noise variance	${\left(20\;\mu g/\sqrt{Hz}\right)}^{2}$	CAI data rate	5 Hz

**Table 2:** Estimation of inertial sensors.

Equipment	Bias Estimation	Actual Value	Relative Error
Gyroscope	0.0097°/h	0.01°/h	3.0%
Accelerometer	41.5625 μg	40μg	3.9%

**Table 3:** Attitude error analysis of the two navigation systems.

	Maximum Error (°)			Reduction (%)		
	Pitch	Roll	Yaw	Pitch	Roll	Yaw
The conventional IMU	−0.010	0.0097	−0.12	—	—	—
Integrated navigation system	−0.011	−0.0079	−0.09	−10.0%	18.6%	25.0%

**Table 4:** Velocity error analysis of the two navigation systems.

	Maximum Error (m/s)		Reduction (%)	
	East	North	East	North
The conventional IMU	1.66	−1.64	—	—
Integrated navigation system	1.42	1.61	14.5%	1.8%

**Table 5:** Position error analysis of the two navigation systems.

	Maximum Error (km)			Reduction (%)		
	Latitude	Longitude	Positioning	Latitude	Longitude	Positioning
The conventional IMU	10.38	40.31	40.39	—	—	—
Integrated navigation system	5.82	27.17	27.34	43.9%	32.6%	32.3%

**Table 6:** Yaw error analysis of different integrated navigation systems.

	Max (°)	Reduction (%)	RMSE (°)	Reduction (%)
The conventional IMU	−0.13	—	0.067	—
Integrated navigation system	−0.09	25.0%	0.052	22.4%
Integrated and drift suppression	−0.09	25.0%	0.051	23.9%

**Table 7:** Position error analysis of different integrated navigation systems.

	Maximum Error (km)			Reduction (%)		
	Latitude	Longitude	Positioning	Latitude	Longitude	Positioning
The conventional IMU	10.38	40.31	40.39	—	—	—
Integrated navigation system	5.82	27.17	27.34	43.9%	32.6%	32.3%
Integrated and drift suppression	5.82	26.72	26.97	43.9%	33.7%	33.2%

## Data Availability

The data presented in this study are available upon request from the corresponding author. The data are not publicly available due to privacy reasons.

## Abbreviations

The following abbreviations are used in this manuscript:

INS	Inertial Navigation Systems.
CAIG	Cold Atom Interferometer Gyroscope.
IMU	Inertial Measurement Unit.
GNSS	Global Navigation Satellite Systems.
CAI	Cold Atom Interferometer.
AGA	Adaptive Gradient Ascent.
MOT	Magneto-optical Trap.
RMSE	Root Mean Square Error.
